# The urgent need for microbiology literacy in society: children as educators

**DOI:** 10.1111/1751-7915.13619

**Published:** 2020-07-10

**Authors:** Kenneth Timmis, James Timmis, Franziska Jebok

**Affiliations:** ^1^ Institute of Microbiology Technical University Braunschweig Germany; ^2^ Athena Institute Vrije Universiteit Amsterdam Amsterdam The Netherlands; ^3^ Uilenstede 510 Amstelveen The Netherlands

## Abstract

Microbes and their activities have pervasive influence and deterministic roles in the functioning and health of the geosphere, atmosphere and biosphere, i.e. in nature. Microbiology can be considered a *language of nature*. We have argued that the relevance of microbes for everyday personal decisions and collective policies requires that society attains microbiology literacy, through the introduction of child‐relevant microbiology topics into school curricula. That is: children should learn the *microbiology language of nature*. Children can be effective transmitters of new and/or rapidly evolving knowledge within families and beyond, where there is a substantive information asymmetry (witness digital technology, social media, and new languages in foreign countries). They can thus be key disseminators of microbiology knowledge, where there will be information asymmetry for the foreseeable future, and thereby contribute to the attainment of microbiology literacy in society. The education of family and friends can be encouraged/stimulated by home assignments, family leisure projects, and school‐organised microbiology‐centric social‐education events. Children are key stakeholders in family decisions. Their microbiology knowledge, and their dissemination of it, can help inform and increase the objectivity of such decisions.

## Graphical abstract

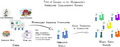

## Children as educators: the case of a foreign language

In an earlier life, one of us (KT) was Head of the Division of Microbiology at the GBF (roughly translated as the National Research Centre for Biotechnology) and HZI (Helmholtz Centre for Infection Research, as the GBF was subsequently renamed) in Braunschweig, Germany, and had the privilege of recruiting, working and developing precious friendships with, and being educated by, many young microbiologists from diverse regions and cultures around the globe. Because the working languages of the group were German and English, learning German was not mandatory. However, for obvious reasons of professional and social interactions, exploration of local delicacies like Harzer Roller, Spargel, Windbeutel, being able to discuss in the local garage the reluctance of the car to fire up on −20°C winter mornings, dating locals, etc., most non‐Germans wanted and tried to learn German.

Almost without exception, those who made the fastest progress and greatest accomplishment in learning the new language and culture idiosyncrasies were parents of young children. Because young children went to Kindergarten and/or school, and in their free time played with other children – both locals and other GBF/HZI children of diverse nationalities and mother tongues who adopted German as their *lingua franca* – they became fluent in German at an astonishing speed. Not only that, German became their preferred language, such that it was common for a fly on the wall to hear family discussions, say in Hindi, punctuated by interjections in German.

This usually transpired 1–2 months after arrival of the family in Braunschweig. After a few more months, family discussions may have been mostly in German, with the occasional interjection in Hindi. The child had unknowingly and without an agenda (though some might remark that children intuitively explore all parental weaknesses in order to get their way, and language disadvantage is a serious weakness in any negotiation) imposed another language on family verbal interactions. This facilitated intenser engagement of the family with locals and the various benefits that this brought, like support when needed, advice on schools and doctors, places to take the children on outings, good garages, shops and restaurants, recipes and how to cook local dishes, as well as more rapid integration into their scientific and local communities. Their children unwittingly functioned as educators and social engineers, acting as shapers of social groups, facilitators of community support and expanders of horizons.

Of course, children do these things anyway – they chatter excitedly and at length at home and social gatherings about things they experienced and that impressed them, positively or negatively, and parents, family and friends absorb (with varying degrees of filtering) the information they communicate. Thus, they educate their families and friends about anything that passionately excites them, in particular things they learned at school from teachers and other children. But their educative activity in regard to a new language for which there is substantial knowledge asymmetry in the family can be particularly striking, because the new language is a key enabler of family adaptation to and integration in a new culture.

## Children as educators in a language of nature: microbiology

We recently stated that *Microbes and their activities have pervasive, remarkably profound and generally positive effects on the functioning, and thus health and well‐being, of human beings, the whole of the biological world, and indeed the entire surface of the planet and its atmosphere. Collectively, and to a significant extent in partnership with the sun, microbes are the life support system of the biosphere. This necessitates their due consideration in decisions that are taken by individuals and families in everyday life, as well as by individuals and responsible bodies at all levels and stages of community, national and planetary health assessment, planning, and the formulation of pertinent policies….We therefore contend that microbiology literacy in society is indispensable for informed personal decisions, as well as for policy development in government and business, and for knowledgeable input of societal stakeholders in such policymaking. An understanding of key microbial activities is as essential for transitioning from childhood to adulthood as some subjects currently taught at school, and must therefore be acquired during general education. Microbiology literacy needs to become part of the world citizen job description* (Timmis *et al*., 2019). In this, we argued that teaching microbiology in schools should have the aim to create future citizens with knowledge and understanding of just those microbial activities that relate to their everyday experiences and the wider issues that affect them and the planet, rather than experts in general microbiology. The task of creating the teaching materials needed for child experience‐centric microbiology curricula is well underway. The first of these has just been published: an article setting out the value of class excursions to explore the manifold examples of microbes and their activities that can be seen and experienced all around us, if we know what and where to seek, and providing a comprehensive list of suggestions for excursions and advice on how to extract maximum benefit from them (McGenity *et al*., 2020).

How long might it take society to attain microbiology literacy? This will obviously vary from country to country, given that formal introduction of microbiology topics into school curricula will occur at different times and speeds in different countries. If we consider only binary teacher:pupil transmission of knowledge, we might conclude that it could take ˜45 years to educate everyone up to the age of 63, somewhat less if we take into account adult education. If we consider the same binary transmission to attain what we might term *effective literacy*, namely a population segment containing a critical mass of decision‐makers and stakeholders exploiting their microbiology literacy to scrutinize the evidence base for, and inform their, decisions on microbiology‐pertinent issues, this might take, say, 20 years. But microbiology knowledge dissemination in society will not be linear and unidirectional, but rather network‐like: a multiplicative and iterative back‐and‐forth of information transfer within families and social groups, catalysed by children and intensified by exciting discoveries reported in the media. This * microbiology knowledge–discovery dissemination network* will accelerate attainment of microbiology literacy in society.

Given that microbes and their activities have pervasive influence and deterministic roles in the functioning and health of the geosphere, atmosphere and biosphere, i.e. of nature, and thereby regulate the health and behaviour of all other organisms and their environments, microbiology might be considered *a language of nature* and because microbiology is foreign to most people, who are unaware of its impact on their daily lives, it may be considered the equivalent of a foreign language. Children can be effective educators of family and friends in the *language of microbiology*.

## Encouragement of family involvement in microbiology education

How can we further support microbiology knowledge transmission in society through our children, especially those who are less communicative? One mechanism would be home assignments, both information research projects1For example, list all the meals your family eats in a week, identify those created by microbes and discuss what the microbes contribute; there is more life under your feet than what you can see: what is it and what is it doing? and especially hands‐on experimental microbiology projects set by the teacher to be run over days or weeks at home,2For example, the set‐up and observation of Winogradsky Columns (Anderson and Hairston, 1999), Mudwatts (Jude and Jude, 2015), visualisation of the small life in house dust, on skin/teeth, in slime in shower basins, on the surface of vegetables, in soil/ponds, on fungal fruiting bodies, etc. with Foldscopes (Cybulski *et al.,* 2014). preferably accompanied by explanatory leaflets/worksheets for the family, that is brief, vividly illustrated texts explaining/depicting the relevance of their children’s projects for their own everyday lives and environment, while emphasizing relevant safety precautions.

And how might we promote active involvement of family in microbiology? This might be achieved by proposing facultative family group discovery projects and local outings (e.g. to water treatment facilities, food production operations, eutrophic ponds/lakes/canals, etc.) – school organized, where necessary – that explore topics of interest to both children and adults, stimulate their curiosity, and trigger discussions about microbial activities and their impact on our lives (Dyer, 2003; McGenity *et al*., 2020). Summer breaks are occasions where families tend to spend more time together and may be ideal periods for trips to locations that offer opportunities for different discoveries, so are perfect for family‐centric microbiology projects (exploring‐discovering microbes on local foods, in beach sand/on pebbles/in rock pools, on boat:water interfaces, historic buildings, etc.). Moreover, schools may consider the possibility of microbiology–themed social events involving children and their families and friends, in the same spirit as sports days, either on‐site or preferably involving excursions of the type we have proposed earlier (McGenity *et al*., 2020), in which some aspect of microbiology is discovered/explored. These could also include events in which external experts from universities, government agencies, industry, etc., are invited to speak about exciting microbiological topics.

The introduction of microbiology curricula in schools and the discovery by children of a previously hidden world, the experience of connecting with a new nature, their bodies’ microbiomes, all the tasty foods created through microbiological processes, sustainability, climate and the atmosphere, the deep sea and subsurface, astrobiology, etc. will ignite a passion for this new *language of nature*. As a consequence of the natural educative activities of children, and additional measures like those listed above to increase family involvement in microbiology‐centric activities, we would argue that the time frame for attainment of microbiology literacy in society is likely to be considerably shorter than might be imagined at first sight.

Interest in acquiring and increasing knowledge of the microbial world will become auto‐amplifying in society once family and friends fully appreciate the importance of microbes for human well‐being, the health of the environment, their centrality in causing, preventing and mitigating crises, such as epi/pandemics, antibiotic resistance, global warming, the soil crisis, the clean water crisis; and how fascinating microbes are and how exciting progress in microbiology knowledge and understanding is. Dare we even say *fashionable* (witness current media interest in the human microbiome), with new topics of conversation at cocktail/dinner parties (*I just read the latest London microbiology story by Martin Adams in the Microbiologist* (https://sfam.org.uk/knowledge/microbiologist‐magazine.html) *and did you know that…?*); regular copy for the press and media (*New source of electricity from poo!*), components of quizzes [*What is the largest living organism on Earth?* (https://www.guinnessworldrecords.com/world‐records/606952‐largest‐living‐organism/)]; ways of impressing friends and colleagues [*I recently learned that we leave a personal signature trail of microbes when we enter a room. Bad news for villains breaking and entering!* (Hampton‐Marcell *et al*., 2017)], etc?

## Children as stakeholders in and influencers of parental‐family decisions

**Figure 1:**
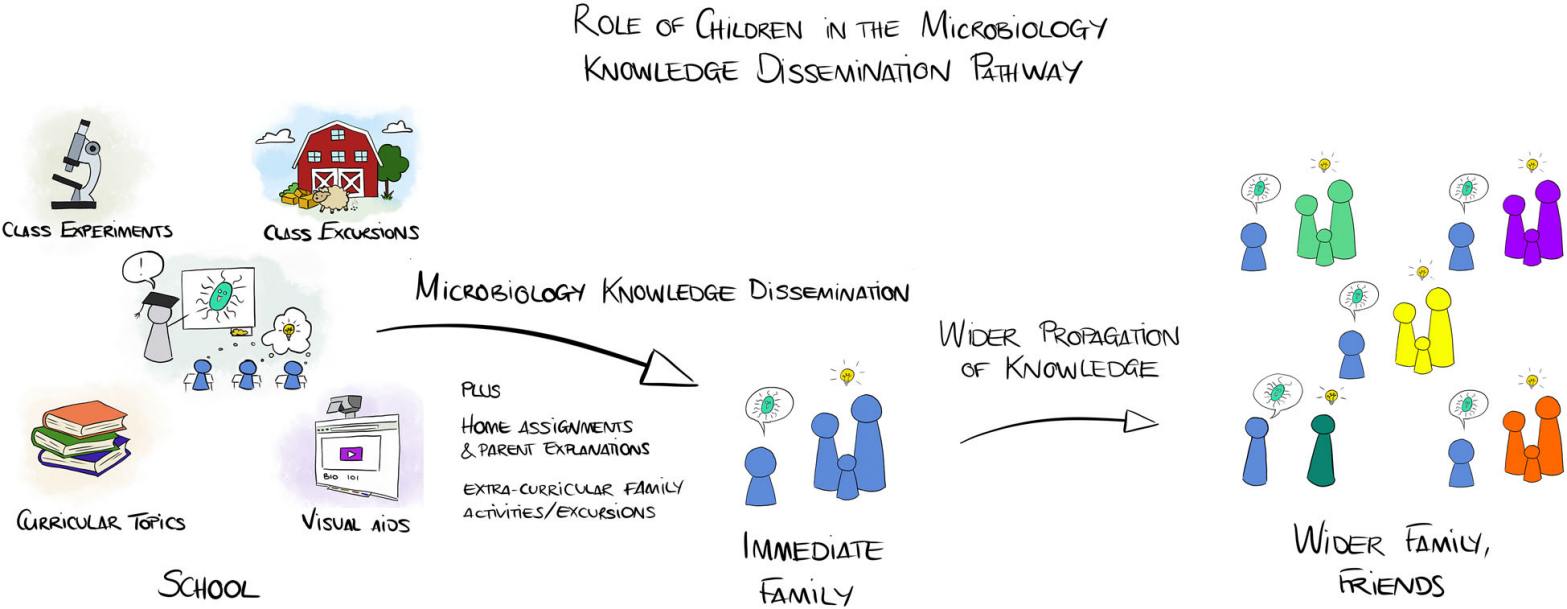
Children can be facilitators and accelerators of microbiology literacy attainment in society. Exciting new information they acquire in school will be transmitted to family and friends. Their role in the microbiology knowledge dissemination pathway can be encouraged and supported by home assignments, family leisure projects, and school‐organised microbiology‐centric social‐education events.

In this context, it is worth drawing attention to another important role of children, namely that as stakeholders in family decisions, especially parental decisions directly affecting them. The educational activity of children may in some cases directly influence the outcome of parental decision‐making, when completeness and quality of information is more important than personal experience and conviction. One example of this is vaccine hesitancy (https://www.who.int/immunization/programmes_systems/vaccine_hesitancy/en/): some vaccine‐hesitant parents may be unsure of the rigorously documented risks and benefits of vaccination, or compelled by ideologically charged narratives rather than scientific arguments and, in consequence, refuse vaccination. If we are able to create at school microbiologically‐literate (and ‐minded) cohorts, these may not only be able to persuade even‐handed parents to reconsider their position *vis‐à‐vis* vaccination, but will, ideally, also mature into adults who critically appraise information and are able to sort the wheat from the chaff. In this day and age of bot‐placed ‘news reports’ and other means of misleading the public, critical appraisal is key (Fig. 1).

Another important example is epi/pandemics, which are not only medical but also societal issues, because public health measures imposed to interrupt infection chains can involve loss of civil liberties (https://www.ncbi.nlm.nih.gov/books/NBK54163/) that go to the heart of our social fabric. Moreover, as experienced with the current SARS‐CoV‐2 pandemic, epi/pandemics can exhibit new, unpredictable characteristics, which are confusing even for public health specialists, and which can engender a bewildering and constantly changing complex plethora of guidelines. During the transition to *effective literacy*, older children in school learning about microbes will more readily understand what is going on, and why and be able to communicate this understanding to the wider world of family and friends. This will, in turn, help counteract misinformation and, ideally, improve coherence with national behavioural strategies and crisis response effectiveness in the population.

In this Editorial, we have argued that children, via interactions with family and friends, will be key facilitators and accelerators of microbiology literacy attainment in society. Increasing awareness of the pervasive impact of microbiological processes on day‐to‐day life, and *vice versa*, is of paramount importance. By providing relevant and, importantly, meaningful (from a child's perspective) and practical school lessons, coupled with exciting class excursions and curiosity‐driven home assignments, we can galvanize children's fascination for day‐to‐day microbiology topics. And by involving their parents and friends in group assignments, we can further support the decrease of information asymmetry between experts in the field and the majority of members in society.

## Conflict of interest

None declared.
